# Graduate Study on the Continent—Austria

**Published:** 1908-11-14

**Authors:** 


### GRADUATE STUDY ON THE CONTINENT—AUSTRIA.

### III.—THE UNIVERSITY OF VIENNA AND ITS COURSES.

THE University of Vienna is the second oldest in the Austrian Empire, having been founded in 1365, some twenty years later than the sister institution at Prague. Of its reputation as a medical school we have already spoken, and it remains only to show what particular benefits it offers to the foreign graduate.

The matriculation fee is 10 kronen. Students must produce satisfactory evidence that they are matriculates of their own University or that they have passed equivalent examinations. Guest tickets are given on application but for regular attendance the usual course fees are to be paid. The fine University library is open daily during term-time, and there are various academic reading-rooms open to students. In the large hospitals there is usually a small reading-room, and the libraries of the various medical societies are available for reference purposes. The large Royal Library is open to readers on application, but will scarcely appeal to the medical graduate. There are many University institutions and clinics which are all open to the graduate on production of his card. For an extended use the usual formalities must be complied with.

These courses are not graduate courses in the sense one usually understands. They are attended by students as well as by qualified men, and for the most part they are elementary. The ordinary lectures are given by the chief professors, which are, of course, attended by students, as well as graduates. In many cases, however, these courses and lectures are very good (we may particularise Professor Lorenz's talks on orthopædic subjects, which are deservedly among the most popular demonstrations at the General Hospital). A few, again, are limited to students. The graduate would do well to obtain the official summary of the lectures, published at the beginning of each term (price 20 heller, or 2d.). Therein he will find particulars of nearly 200 courses and lectures, varying from 4 kronen upwards, and will be able to make his selection at leisure. If, however, he expects to find the courses equal to those which are given at Berlin, Hamburg, or Leipsig, he will be disappointed. As we have stated, they are elementary for the most part, and though the material is large it is not fully used. Operative courses are good, and practical courses with limited membership equally so; but it must always be remembered that these are open to the objections which can be drawn up against mixed courses. The fees are not much lower than those at other centres.

### THE AMERICAN MEDICAL ASSOCIATION OF VIENNA.

Like Berlin, Vienna has its association of English-speaking graduates. This is the American Medical Association of Vienna, which was founded in 1903. Since then the society has flourished, and is at present a fairly strong one.

Its objects, as set forth in the modified constitution adopted in October 1907, are "to promote the social intercourse and the scientific advancement of its members, to provide information in regard to the scope and relative value of courses, and to furnish data for the rapid orientation of new members in regard to pensions, rooms, restaurants, etc." "All physicians of good standing are eligible to membership." The executive consists of a President (Dr. Paul Waterman), a Vice-President (Dr. Stuever), and a Secretary and Treasurer (Dr. R. Fletcher), an Executive Committee of three members, and an Orientation Committee of seven members, who have to deal with seven special subjects: internal medicine, surgery, gynæcology and obstetrics, pathology, ophthalmology, otology, pediatrics, and genito-urinary diseases. The life membership fee is 10 kronen. Regular meetings of the society are held at least every two weeks, except during July and August.

The society issues a tiny little blue book containing its constitution and by-laws, a summary of special courses, lists of hotels and pensions, and a few notes of interest to newcomers. The price of this little pamphlet is 1 crown. The rules governing the "book courses," or courses given directly under the auspices of the society, are given in this blue book. On turning to the book one finds that the majority of them are University courses such as have already been mentioned, and which need not therefore be further discussed here. In the majority of these the fees are high, and nothing is offered which the graduate cannot obtain by attaching himself to the ordinary courses.

The headquarters of the Association are at the Café Zür Klinik, corner of Spital and Lazarethgasse, a small general café opposite the side entrance to the Allgemeines Krankenhaus. Here the lists are posted, American papers are to be found, and the members meet in social intercourse. The regular fortnightly meetings of the society are held in the lecture-theatre of the psychiatric clinic—the late Professor Krafft-Ebing's lecture-room.

It is perhaps unfair—perhaps also unwise—to attempt comparisons, but the differences between the Berlin and Vienna Associations are so great that one cannot pass them over in silence. To begin with, one misses entirely the spirit of good fellowship which makes the Berlin meetings at the Heidelberger Café such enjoyable functions. The Vienna society has reversed the process. Its meetings are dull and held in seclusion; its headquarters are at a public café, where it is impossible to read in quiet, and where one is always under the obligation of having to order something.

The Berlin headquarters, on the other hand, are at a professional reading-room, where members can quietly read or talk remote from the distractions of café life. The student will not find any difference between the members. They are courteous, kindly gentlemen, as we have always found the American post-graduate student to be, willing to give the novice the best of advice and the most useful tips. These, however, he may obtain without joining the society; and at present it appears to us that the Association adopts a too exclusive and narrow policy to make the English student feel comfortable as a member. We regret this the more as we feel sure that such an association can do a great deal of good at a centre like Vienna, and we can only express the hope that it may take a leaf out of the book of its Berlin counterpart.

We do not for a moment wish to belittle the good that the Association has done and which it is still doing. What we wish to point out, however, is that the student can get along very well in Vienna without being a member of the society. The average course at Vienna, for instance, compares very unfavourably with the average Berlin course. It is more elementary, more adapted to students than to post-graduates. As we have pointed out when dealing with German courses, there is a tendency in some quarters to regard the graduate student as wanting in elementary knowledge, and to lecture or demonstrate accordingly. It is, of course, the fault of the student that this tendency is on the increase, and we are glad to state that we have personally heard American students protest against such elementary courses, which are directly against the spirit of graduate teaching.

### The Hospitals of Vienna.

The following is a list of the chief hospitals and clinical institutions of the city :—

K. k. Allgemeines Krankenhaus, IX. Alserstrasse 4. K. k. Krankenhaus (Wieden), IV. Favoritenstrasse 30-32. K. k. Krankenhaus (Rudolfsstiftung), III. Rudolfsgasse 15. K. k. Franz Joseph-Spital und Diphtherie-Serum-Lab., X. Kundratstrasse 3. Lupusheilstatte, IX. Borschkegasse 10. K. k. Kaiserin Elisabeth-Spital, XIV. Huglgasse 3. K. k. Kronprinzessin Stephanie-Spital, XVI. Thaliastrasse 44. K. k. Wilhelminen-Spital, XVI. Montleartstrasse 1. N. ö. Findelanstalt, VIII. Alserstrasse 21-23. N. ö. Landes-Irrenanstalt, IX. Lazarethgasse 14. Erzherzogin Sophien-Spital, VII. Kaiserstrasse 7. Karolinen-Kinderspital, IX. Schubertgasse 23. Erstes Kinderspital "Zur heiligen Anna," IX. Kinderspitalgasse 6. Rothschild-Spital, XVIII. Währingergürtel 97. Rudolfinerhaus, Hospital and Training school for Nurses, XIX. Billrothstrasse 78. Poliklinik-Spital, IX. Mariannengasse 10. Franz Joseph-Ambulatorium, VI. Sandwirthgasse 3. Normal Anatomical Museum, IX. Währingerstrasse 13. Military Anatomical Collection, IX. Währingerstrasse 25. Pathological Anatomical Museum, Pathological Institut Allg. Krankenhaus. National Institute for Serotherapy and Therapy of Hydrophobia; Rudolf Hospital. Pathological Institute; Wilhelminen Hospital. Hospital for Tuberculosis, Alland bei Baden. National Institute for the Deaf and Dumb, IV. Favoritenstrasse 13.

With some of these we shall deal later. Suffice it for the present to say that the student will find something of interest in all of them, and that to exhaust the material which is available in these institutions is practically an impossibility.

### Medical and Social Associations.

There are a large number of medical and professional associations and societies at Vienna. Particulars of meetings, etc., the student will find in the weekly issues of the *Wiener Medizinische Wochenschrift*, the main medical paper of the city. For information regarding membership of the following societies, application should be made directly to the Secretaries: Laryngological Society; Medical Society; Dermatological Society; Royal Society of Physicians (IX. Frankengasse 8); Neuro-Psychiatrical Society; Obstetrical Society. The influential American Medical Association has its headquarters at the Café Klinik, corner of Spital and Lazarethgasse IX. In the next article we will deal more fully with the University clinics and institutions.

The Anglo-American Society of Vienna is a purely social Club, the headquarters of which are at the Imperial Hotel, I. Kärntnerring 16. It was founded in December 1907. An influential general committee has been formed, and the society appears to be flourishing. It has undoubtedly an excellent programme, and is worth the cordial support of every English-speaking visitor to Vienna. The membership fee is quite small, and the society is a practical and not a merely *dilletanti* association.

For the student who desires to be in constant attendance at the clinics, a lodging in the "clinical quarter," the IXth district (Alsersgrund) is to be recommended. Rooms, either *en garçon* or at a pension, are easily obtainable here; those in Alserstrasse or the streets leading out of it are to be recommended. A bed-sitting-room is usually provided, though the charges are relatively high. A bedroom with separate sitting-room is much more difficult to obtain in this quarter, and the rents charged are exorbitant. The pensions are all fair, and their number is legion. The student is advised not to trust to recommendations, but to inspect things for himself before he finally decides on a lodging. This will save unnecessary changes and their attendant discomforts and inconveniences.

